# Physical triggers in takotsubo syndrome: a high-risk phenotype? insights from the eVOLUTION registry

**DOI:** 10.1093/ehjci/jeag017

**Published:** 2026-02-05

**Authors:** Riccardo Cau, Julian Luetkens, Gianluca Pontone, Giuseppe Muscogiuri, Riccardo Faletti, Roberta Montisci, Luca Arcari, Sebastien Normant, Federica Catapano, Tommaso D’Angelo, Leon Bischoff, Antonio Esposito, Anna Palmisano, Antonella Meloni, Federica Ciolina, Francesco Negri, Costanza Lisi, Massimo Imazio, Maria Francesca Marchetti, Nicola Galea, Alessandra Volpe, Alfredo Blandino, Giacomo Pambianchi, Alberto Clemente, Jean Nicolas Dacher, Marco Gatti, Luca Saba, EVOLUTION group, Cosimo Agrimi, Luca Arcari, Francesco Balata, Leon Bischoff, Alfredo Blandino, Federica Catapano, Riccardo Cau, Federica Ciolina, Alberto Clemente, Jean Nicolas Dacher, Tommaso D’Angelo, Fabrizio D’Ascenzo, Antonio Esposito, Riccardo Faletti, Nicola Galea, Marco Gatti, Massimo Imazio, Costanza Lisi, Julian Luetsken, Maria Francesca Marchetti, Gloria Marras, Antonella Meloni, Roberta Montisci, Giuseppe Muscogiuri, Francesco Negri, Anna Palmisano, Giacomo Pambianchi, Alessandro Pinna, Laura Pistoia, Francesco Pisu, Gianluca Pontone, Luca Saba, Normant Sebastien, Giulio Antonino Strazzarino, Alessandra Volpe, Benedetta Volpi

**Affiliations:** Department of Radiology, Azienda Ospedaliero-Universitaria (A.O.U.) di Cagliari – Polo di Monserrato s.s. 554 km 4.500, Monserrato, Cagliari 09045, Italy; Department of Diagnostic and Interventional Radiology, University Hospital Bonn, Venusberg-Campus 1, Bonn 53127, Germany; Perioperative Cardiology and Cardiovascular Imaging Department, Centro Cardiologico Monzino IRCCS, Via C. Parea 4, Milan 20138, Italy; Department of Biomedical, Surgical and Dental Sciences, University of Milan, Milan, Italy; Department of Radiology, ASST Papa Giovanni XXIII, Bergamo, Italy; Department of Surgical Sciences, University of Turin, Turin, Italy; Radiology Unit - Candiolo Cancer Institute-FPO-IRCCS, Candiolo, Italy; Department of Cardiology, Azienda Ospedaliero-Universitaria (A.O.U.) di Cagliari – Polo di Monserrato s.s. 554, Monserrato, Cagliari 09045, Italy; Cardiology Unit, Madre Giuseppina Vannini Hospital, Rome, Italy; Medical Imaging Department, CHU Rouen, Rouen F-76000, France; Department of Biomedical Sciences, Humanitas University, via Rita Levi Montalcini 4, Milan 20090, Pieve Emanuele, Italy; Department of Radiology, IRCCS Humanitas Research Hospital, via Manzoni 56, Milan, Rozzano 20089, Italy; BIOMORF Department, Diagnostic and Interventional Radiology Unit, University Hospital “AOU G. Martino,” Via Consolare Valeria 1, Messina 98100, Italy; Department of Diagnostic and Interventional Radiology, University Hospital Bonn, Venusberg-Campus 1, Bonn 53127, Germany; Advanced Imaging for Personalized Medicine Unit, Experimental Imaging Center, IRCCS San Raffaele Hospital, Milan, Italy; School of Medicine, Vita-Salute San Raffaele University, Milan, Italy; Advanced Imaging for Personalized Medicine Unit, Experimental Imaging Center, IRCCS San Raffaele Hospital, Milan, Italy; School of Medicine, Vita-Salute San Raffaele University, Milan, Italy; Department of Radiology, Fondazione G. Monasterio CNR-Regione Toscana, Pisa, Italy; Cardiology Unit, Madre Giuseppina Vannini Hospital, Rome, Italy; Cardiothoracic Department, University Hospital “Santa Maria Della Misericordia,” Azienda Sanitaria Universitaria Integrata Friuli Centrale, Udine, Italy; Department of Biomedical Sciences, Humanitas University, via Rita Levi Montalcini 4, Milan 20090, Pieve Emanuele, Italy; Department of Radiology, IRCCS Humanitas Research Hospital, via Manzoni 56, Milan, Rozzano 20089, Italy; Cardiothoracic Department, University Hospital “Santa Maria Della Misericordia,” Azienda Sanitaria Universitaria Integrata Friuli Centrale, Udine, Italy; Department of Medicine, University of Udine, Udine, Italy; Department of Cardiology, Azienda Ospedaliero-Universitaria (A.O.U.) di Cagliari – Polo di Monserrato s.s. 554, Monserrato, Cagliari 09045, Italy; Department of Radiological, Oncological and Pathological Sciences, Sapienza University of Rome, Rome 00161, Italy; Perioperative Cardiology and Cardiovascular Imaging Department, Centro Cardiologico Monzino IRCCS, Via C. Parea 4, Milan 20138, Italy; BIOMORF Department, Diagnostic and Interventional Radiology Unit, University Hospital “AOU G. Martino,” Via Consolare Valeria 1, Messina 98100, Italy; Department of Radiological, Oncological and Pathological Sciences, Sapienza University of Rome, Rome 00161, Italy; Department of Radiology, Fondazione G. Monasterio CNR-Regione Toscana, Pisa, Italy; Medical Imaging Department, CHU Rouen, Rouen F-76000, France; Department of Radiology, ASST Papa Giovanni XXIII, Bergamo, Italy; Radiology Unit, AOU Città Della Salute e Della Scienza di Torino – Molinette Hospital, Turin, Italy; Department of Radiology, Azienda Ospedaliero-Universitaria (A.O.U.) di Cagliari – Polo di Monserrato s.s. 554 km 4.500, Monserrato, Cagliari 09045, Italy

**Keywords:** takotsubo syndrome, cardiovascular magnetic resonance, physical trigger, prognosis

## Abstract

**Background:**

Physical triggers (PT) are increasingly recognized as important determinants of outcomes in Takotsubo syndrome (TS). This multicenter study investigated the prevalence, clinical features, cardiovascular magnetic resonance (CMR) findings, and prognostic impact of PT in patients with TS.

**Methods and results:**

In this retrospective registry, 399 TS patients (mean age 70.1 ± 11.8 years, 91% female) were included with a median follow-up of 26.7 months. A PT was identified in 30.5% of cases, an emotional trigger in 38.8%, and no trigger in 30.5%. Patients with PT showed higher C-reactive protein levels (*P* = 0.008), lower troponin values (*P* = 0.018), less frequent and less extensive T2-STIR abnormalities (*P* = 0.007 and *P* = 0.005, respectively) and LGE (*P* = 0.002 and *P* = 0.005, respectively), longer hospital stays (*P* = 0.002), and more frequent in-hospital complications (*P* = 0.001). Kaplan–Meier analysis demonstrated significantly lower event-free survival in the PT group compared with patients in the emotional or no-trigger groups (log-rank *P* = 0.003). In multivariable Cox regression analysis, the presence of a physical trigger (*P* = 0.037) and pre-existing neurological disease (*P* = 0.027) were independently associated with a higher risk of all-cause mortality and post-discharge adverse events.

**Conclusion:**

TS patients with PT represent a high-risk subgroup with worse in-hospital outcomes and increased post-discharge events. Careful identification of the trigger type may therefore help stratify risk, allowing for closer monitoring during hospitalization and more vigilant long-term management in the outpatient setting.

## Graphical Abstract

**Graphical Abstract:**
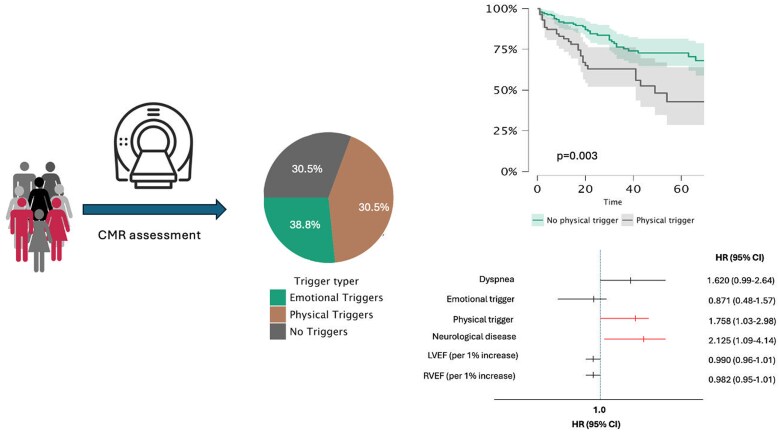

## Introduction

Takotsubo syndrome (TS) is an acute and reversible form of heart failure, predominantly affecting post-menopausal women, and is characterized by transient left ventricular (LV) systolic dysfunction.^1–4^ Traditionally considered a benign condition due to the rapid recovery of myocardial function and generally favourable outcomes, recent evidence has challenged this perception.^5–11^ Identifying clinical parameters that can predict outcomes in TS is therefore essential. Although the underlying mechanisms remain incompletely understood, both somatic diseases and emotional events have been recognized as potential triggers^1,12^ and emerging data suggest that the type of trigger may influence clinical presentation and patient outcomes. For instance, data from the multicenter German–Italian–Spanish (GEIST) Registry have shown that patients with an emotional trigger experience lower rates of in-hospital complications and reduced long-term mortality compared with those with a physical or no trigger.^13^ Nevertheless, data on trigger-associated clinical presentation, cardiovascular magnetic resonance (CMR) findings, and outcomes remain limited. In this multicenter study, we aimed to investigate the prevalence, clinical correlates, CMR characteristics, and prognostic impact of physical triggers in patients with TS undergoing CMR.

## Methods

### Study population

The rationale and design of the EVOLUTION Registry have been previously described in detail.^7^ In brief, EVOLUTION was a retrospective, multicenter study of patients with a diagnosis of TS who fulfilled the criteria outlined in the Position Statement of the Heart Failure Association of the European Society of Cardiology and was referred for clinical CMR imaging. Diagnostic criteria include regional wall motion abnormalities extending beyond a single epicardial vascular territory, typically preceded by a stressful trigger, in the absence of culprit atherosclerotic disease as assessed by invasive coronary angiography. Additional criteria comprise new electrocardiographic abnormalities, elevated serum natriuretic peptides with an increase in cardiac troponin levels, and recovery of left ventricular dysfunction at follow-up.

Between November 21, 2007, and December 22, 2024, consecutive patients who underwent completed CMR studies at 10 sites were enrolled. Exclusion criteria were applied as previously described.^7^ In the current study, patients were divided into two subgroups: pT and TS with either an emotional or no identifiable trigger. Trigger classification was performed retrospectively at each participating centre by investigators blinded to imaging and outcome data. Participants with a potential combination of physical and emotional triggers that could not be clearly distinguished, as well as those with missing data on the trigger mechanism, were excluded from the analysis.

CMR studies were performed at each participating centre according to clinical indication and locally approved imaging protocols.^14,15^

CMR protocols included mandatory sequences: cine imaging in short- and long-axis views, T2-weighted imaging in short- and long-axis views, and LGE imaging in short- and long-axis views. The study was approved by local institutional review boards in accordance with the Declaration of Helsinki., with a waiver of written informed consent.

### CMR image post-processing

CMR data were anonymized before analysis and transferred to commercially available software for analysis (CVI42, version 6.2; Circle Cardiovascular Imaging Inc., Calgary, Canada). Images were interpreted by an experienced observer with over 10 years of expertise in cardiovascular imaging, who was blinded to all clinical data and outcomes. All quantitative measurements of left and right ventricular volumetric parameters were performed in a dedicated core laboratory, in accordance with recommendations from the Society for CMR and the European Association of Cardiovascular Imaging.^14,15^ Endocardial and epicardial borders of both ventricles were manually contoured on short-axis cine images at end-diastole and end-systole. Papillary muscles and trabeculae were included in the ventricular cavity and excluded from myocardial mass calculations. End-diastolic volume (EDV), end-systolic volume (ESV), and stroke volume (SV) were calculated using the summation-of-disks method (Simpson’s rule), and ejection fraction (EF) was derived as (EDV–ESV)/EDV×100%. Left and right ventricular masses were indexed to body surface area to obtain LV and RV mass indices. RV dysfunction was defined as an RV ejection fraction below sex-specific reference values (<44% in men and <47% in women) as assessed by CMR, according to current normal reference ranges.^16^ LV dysfunction was defined as an LV ejection fraction below 50% as assessed by CMR.^17^

Focal areas of regional high signal intensity with a non-ischaemic distribution pattern were visually assessed on T2-weighted short-tau inversion recovery (T2-STIR) and late gadolinium enhancement (LGE) images, using short-axis ECG-gated steady-state free-precession cine images as reference and confirmed in two perpendicular views. The extent of T2-STIR hyperintensity and LGE was then evaluated semiquantitatively as the number of segments showing a non-ischaemic LGE pattern, according to the 17-segment model of the American Heart Association (AHA).^18^

### Definition of in-hospital complications and post-discharge adverse events

All patients were followed up through clinical visits after the CMR examinations, and hospital records were reviewed for clinical events by clinicians at each participating site.

In-hospital complications were defined as major cardiovascular and cerebrovascular adverse events occurring during hospitalization, including all-cause mortality, pulmonary oedema, arrhythmias, cardiogenic shock, transient ischaemic attack, and ischaemic stroke. The primary endpoint was a composite of all-cause mortality and major cardiovascular or cerebrovascular adverse events, including heart failure hospitalization, arrhythmias, recurrence, transient ischaemic attack, and ischaemic stroke.

Pulmonary oedema was defined as the presence of respiratory distress and pulmonary rales due to pulmonary congestion, confirmed by chest radiography, respiratory failure (hypoxaemia-hypercapnia), tachypnea (>25breaths/min), and increased work of breathing.^19^

Arrhythmia was defined as the presence of asystole, pulseless electrical activity, complete sinoatrial or atrioventricular block, new-onset atrial fibrillation, ventricular tachycardia, or ventricular fibrillation.^20^

Cardiogenic shock was defined as a sustained systolic blood pressure below 90 mm Hg for at least 30 min or the need for vasopressors, inotropes or mechanical circulatory support to maintain systolic blood pressure ≥90 mm Hg, accompanied by clinical signs of pulmonary congestion and impaired organ perfusion, the latter evidenced by at least one of the following: (i) altered mental status; (ii) cold, clammy skin or extremities; (iii) oliguria (urine output ≤30 mL/h); or (iv) an arterial lactate concentration ≥2 mmol/L (corresponding to ≥18 mg/dL).^21^

Recurrence of TS was defined as the occurrence of new wall motion abnormalities in the absence of obstructive coronary artery disease, after complete recovery from the index TS event.^22^

Stroke was defined as an ischaemic cerebral infarction due to embolic or thrombotic occlusion of a major intracranial artery.^23^ Transient ischaemic attack was defined as the sudden onset of focal neurological signs or symptoms that resolved within 24 h.^24^

### Statistical analysis

Continuous variables are presented as mean ± standard deviation (SD) or median (interquartile range, IQR), as appropriate. Normality of distributions was assessed using the Kolmogorov–Smirnov test. Group comparisons of continuous variables were performed with the independent-samples *t* test for normally distributed data and the Mann–Whitney *U* test for non-normally distributed data. Categorical variables are expressed as counts and percentages, and were compared using the chi-square test or Fisher’s exact test, as appropriate. Comparisons reported were considered descriptive; therefore, these results should be interpreted as exploratory.

Event-free survival was evaluated using Kaplan–Meier estimates, with differences between groups assessed by the log-rank test.

Cox proportional hazards regression models were applied to examine the association between trigger type and outcomes. The exposure variable was the type of trigger, categorized into three groups: *Physical triggers*, *Emotional triggers*, and *No triggers*. Dummy coding was applied with *Emotional triggers* as the reference category, allowing estimation of hazard ratios (HRs) and 95% confidence intervals (CIs). As a secondary analysis, a binary variable was created to compare *Physical triggers* vs. *non-Physical triggers.*

Univariable Cox proportional hazards regression was used to identify predictors of the primary endpoint. Variables with a *P* value < 0.05 in univariable analysis were included in the initial multivariable model. This model comprised 6 variables with 75 events, resulting in an events-per-variable (EPV) of 12.5, A second multivariable model was then constructed including the same univariable predictors along with clinically relevant covariates—age, sex, left ventricular ejection fraction, troponin, C-reactive protein (CRP), and hospitalization duration—resulting in 11 variables and an EPV of 6.8. Variables with evidence of collinearity (Spearman’s *ρ* ≥ 0.7) were excluded to maintain model stability.

Proportional hazards assumptions were verified by Schoenfeld residuals. HRs are reported with 95% CIs. All tests were two-tailed, and a *P* value < 0.05 was considered statistically significant. Statistical analyses were performed using JASP.

## Results

### Baseline characteristics

**Figure 1:**
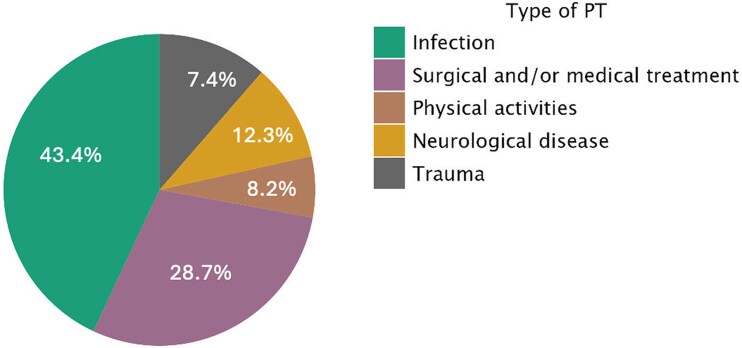
Pie chart illustrating the distribution of different types of PT. Each sector represents the relative frequency of a specific physical trigger identified in the study population.

A total of 399 patients with TS were included in the analysis, comprising 364 females (91.2%) and 35 males (8.8%), with a mean age of 70.1 ± 11.8 years. Baseline characteristics of the overall study population are summarized in *Table 1*. Among the 399 patients included in this multicenter study, a PT was identified in 122 patients (30.5%), an emotional trigger in 155 patients (38.8%), while no trigger was observed in 122 patients (30.5%). Specifically, PTs included 53 cases (43.4%) of infection, 35 (28.7%) medical and/or surgical interventions, 10 (8.2%) physical activities, 15 (12.3%) neurological diseases, and 9 (7.4%) traumatic events. *Figure 1*.

**Table 1:** Baseline and CMR characteristics of patients with TS

Variables	Overall (*n* = 399)	No physical trigger (*n* = 277)	Physical trigger (*n* = 122)	*P*-value
Age, mean ± SD	70.11 ± 11.8	70.69 ± 10.7	68.80 ± 14.1	0.192
Sex (Male), *n* (%)	35 (8.7)	20 (7.7)	15 (12.2)	0.110
BSA, mean ± SD	1.72 ± 0.1	1.72 ± 0.2	1.70 ± 0.17	0.480
BPM, mean ± SD	81.21 ± 20	80.17 ± 19.2	83.77 ± 21.5	0.118
Troponin T, mean ± SD	1824.61 ± 3161.5	1988.08 ± 2983.5	1469.84 ± 3522.5	**0**.**018**
Troponin I, mean ± SD	4.18 ± 5.2	4.71 ± 5.6	3.19 ± 4.5	0.132
proBNP, mean ± SD	4992.72 ± 9084.81	5434.23 ± 10344	3937.25 ± 4817.6	0.269
C-reactive Protein, mean ± SD	26.91 ± 44.5	22.45 ± 36.7	38.65 ± 58.36	**0**.**008**
Hypertension, *n* (%)	252 (63.1)	184 (66.9)	68 (55.2)	0.023
Dyslipidemia, *n* (%)	202 (50.6)	147 (53.4)	55 (44.7)	0.100
Obesity, *n* (%)	56 (14)	41 (14.9)	15 (12.1)	0.458
Smoke, *n* (%)	70 (17.7)	44 (16)	26 (21.1)	0.707
Diabetes, *n* (%)	61 (15.2)	42 (15.2)	19 (15.4)	0.976
CAD, *n* (%)	55 (13.7)	39 (14.1)	16 (13)	0.724
COPD, *n* (%)	33 (8.2)	18 (6.5)	15 (12.1)	0.061
Malignancies, *n* (%)	74 (18.5)	48 (17.4)	26 (21.3)	0.393
Neurological disease, *n* (%)	44 (11)	26 (9.4)	18 (14.6)	0.132
Psychiatric disease, *n* (%)	52 (13)	37 (13.4)	15 (21.1)	0.743
Typical chest Pain, *n* (%)	231 (57.8)	184 (66.9)	47 (38.1)	**< 0.001**
Dyspnoea, *n* (%)	140 (35)	92 (33.4)	48 (39)	0.299
T-wave inversion, *n* (%)	153 (38.3)	118 (42.9)	34 (27.6)	**0**.**025**
ST-segment elevation, *n* (%)	86 (21.5)	65 (23.3)	20 (16.2)	0.275
Corrected QT interval, mean (SD)	502.79 (86.03)	508.25 (84.1)	474.14 (92.8)	0.150
Duration of hospitalization, mean (SD)	9.46 (8.3)	8.50 (5.8)	12.51 (12.9)	**0**.**002**
In hospital complications, *n* (%)	87 (21.8)	48 (17.4)	39 (31.7)	**0**.**001**
Out-of-hospital complications, *n* (%)	75 (18.7)	43 (15.6)	32 (26)	**0**.**014**
LV Apical ballooning, *n* (%)	292 (73.1)	197 (71.6)	95 (76.8)	0.424
Mid-ventricular ballooning, *n* (%)	50 (12.5)	34 (12.3)	16 (13)	0.884
Basal ballooning, *n* (%)	15 (3.7)	8 (2.9)	7 (5.6)	0.185
Focal ballooning, *n* (%)	26 (6.5)	19 (6.9)	7 (5.6)	0.615
Biventricular ballooning	71 (16.3%)	45 (16.3%)	26 (21.1)	0.215
LVEF, mean ± SD	47.91 ± 11.86	47.94 ± 11.8	47.76 ± 12.2	0.886
EDV LV, mean ± SD	128.46 ± 42.33	128.78 ± 44.9	127.5 ± 36.1	0.785
ESV LV, mean ± SD	66.48 ± 31.18	66.58 ± 32.6	66.29 ± 32.6	0.931
SV LV, mean ± SD	61.32 ± 22.25	61.31 ± 22.5	61.09 ± 21.5	0.926
RV involvement	71 (16.3%)	45 (16.3%)	26 (21.1)	0.215
RVEF, mean ± SD	53.63 ± 9.21	54.27 ± 9.1	52.16 ± 9.3	**0**.**037**
EDV RV, mean ± SD	109.35 ± 33.48	107.36 ± 32.7	113.4 ± 34.6	0.100
ESV RV, mean ± SD	52.45 ± 31.12	51.24 ± 33.9	55 ± 23.6	0.274
SV RV, mean ± SD	59.63 ± 19.60	59.42 ± 19.4	59.87 ± 19.9	0.836
Time CMR, mean ± SD	7.37 ± 8.96	7.61 ± 9.1	6.82 ± 8.6	0.722
T2 STIR, *n* (%)	301 (75.4)	219 (79)	82 (66.6)	**0**.**007**
T2 STIR (segments involvement), mean ± SD	4.12 ± 3.22	4.42 ± 3.2	3.44 ± 2.9	**0**.**005**
LGE, *n* (%)	75 (18.7)	63 (22.9)	12 (9.7)	**0**.**002**
LGE (segments involvement), mean ± SD	1.14 ± 2.52	1.42 ± 2.7	0.48 ± 1.92	**0**.**005**
Thrombus, *n* (%)	5 (1.2)	2 (0.7)	3 (2.4)	0.156
Pericardial effusion, *n* (%)	120 (30)	74 (26.9)	46 (37.3)	**0**.**045**
Pleural effusion, *n* (%)	88 (22)	49 (17.6)	39 (31.7)	**0**.**001**

Numbers in bold type indicate a significant difference.

BPM, Beats Per Minute; BSA, body surface area; CAD, coronary artery disease; CMR, cardiovascular magnetic resonance; COPD, Chronic Obstructive Pulmonary Disease; EDV, end-diastolic volume; ESV, end-systolic volume; LGE, late gadolinium enhancement; LV, left ventricle; LVEF, left ventricular ejection fraction; RA, right atrium; RV, right ventricle; RVEF, right ventricular ejection fraction; SD, standard deviation; STIR, short tau inversion recovery; SV, stroke volume.

No significant differences were observed between patients without and with PT in terms of sex, age, or comorbidities (*Table 1*). Patients with PT showed a lower prevalence of hypertension compared with those with emotional or no trigger (55.2% vs. 66.9%, *P* = 0.023). No other differences were noted regarding cardiovascular risk factors. Patients without PT more frequently reported chest pain (66.5% vs. 38.1%, *P* < 0.001). No significant differences were detected for corrected QT interval or the presence of ST-segment elevation. However, patients with PT had a lower prevalence of T-wave inversions (27.6% vs. 42.9%, *P* = 0.025).

Regarding TS variants, no significant differences were observed among patients with different triggers. The PT group demonstrated lower troponin *T* values (1469.84 vs. 1988.08 ng/L, *P* = 0.018) and higher CRP levels (38.65 vs. 22.45 mg/L, *P* = 0.008) compared with patients with emotional or no trigger. Moreover, patients with PT experienced a longer duration of hospitalization (12.51 vs. 8.50 days, *P* = 0.002).

### Cardiovascular magnetic resonance findings

On CMR, no significant differences were observed between patients with and without PT in terms of LV ejection fraction (EF) or end-diastolic and end-systolic volumes. Similarly, RV volumes did not differ significantly, although patients with PT had a slightly lower RV EF compared with those without PT (52.2% vs. 54.3%, *P* = 0.037).

Tissue characterization revealed marked differences between groups. Patients with PT showed a lower prevalence of myocardial oedema on T2-STIR imaging (66.6% vs. 79.2%, *P* = 0.007) and less extensive oedema (3.4 ± 2.9 vs. 4.4 ± 3.2 segments, *P* = 0.005). Likewise, LGE was less frequent in the PT group (9.7% vs. 22.9%, *P* = 0.002) and, when present, involved fewer segments (0.48 ± 1.9 vs. 1.42 ± 2.7, *P* = 0.005). In both groups, when LGE was observed, the most common pattern consisted of a faint, patchy enhancement with minimal transmurality, typically located in segments exhibiting wall motion abnormalities.

To ensure these findings were not influenced by the timing of imaging, we performed a subgroup analysis stratified by CMR timing (≤5 days vs. > 5 days), confirming that the lower prevalence of oedema on T2-STIR imaging and LGE in the PT group remained statistically significant even among patients who underwent early CMR (79.4% vs. 92.5%, *P* = 0.002 and 12.7% vs. 26%, *P* = 0.032, respectively).

Pericardial effusion was more common in patients with PT (37.3% vs. 26.9%, *P* = 0.045), as was pleural effusion (31.7% vs. 17.4%, *P* = 0.001). No significant differences were found regarding the presence of LV thrombus (2.4% vs. 0.7%, *P* = 0.156).

### In-hospital complications and post-discharge adverse events

**Figure 2:**
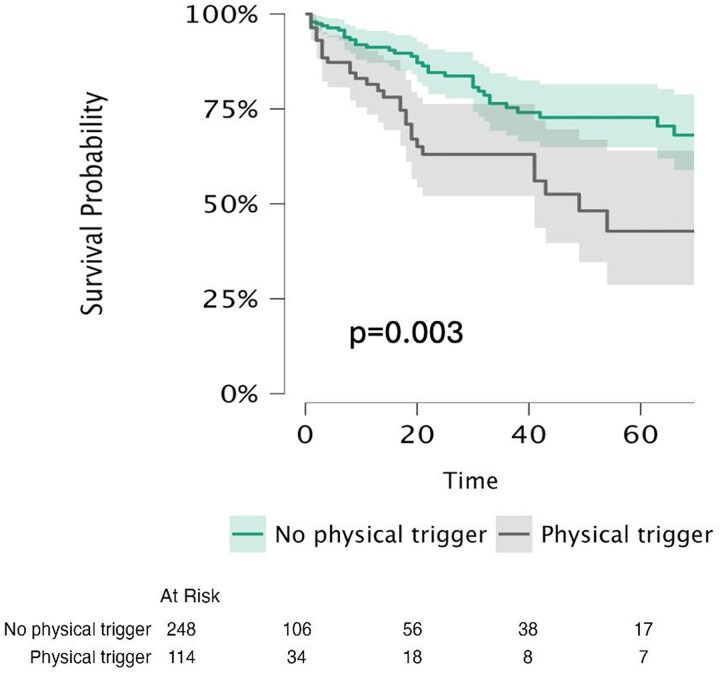
Kaplan–meier curves comparing patients with TS with and without physical trigger, illustrating differences in long-term all-cause mortality and post-discharge adverse events.

In-hospital complications occurred in 87 patients (21%) and included pulmonary oedema in 37 patients (9%), arrhythmias in 31 patients (8%), cardiogenic shock in 14 patients (3.5%), death in 3 patients (1%), transient ischaemic attack in 1 patient (<1%), and ischaemic stroke in 1 patient (<1%). TS patients with PT had a significantly higher incidence of in-hospital adverse events compared with those with an emotional or no trigger (31.7% vs. 17.4%; *P* = 0.002). Over a median follow-up of 26.7 months (interquartile range, 3–37 months), 75 patients (19%) experienced post-discharge adverse events, including 22 hospitalizations for heart failure (5%), 20 recurrences of TS (5%), 15 deaths (4%), 14 arrhythmias (3%), 3 transient ischaemic attacks (1%), and 1 stroke (<1%). All-cause mortality occurred in 3 patients with an emotional trigger (20%), 7 patients with a physical trigger (47%), and 5 patients without an identifiable trigger (33%). The remaining 324 patients (81.2%) completed follow-up without events. The primary outcome (a composite of all-cause mortality and post-discharge adverse events) was observed in 15.6% of patients without PT compared with 26% of those with PT (*P* = 0.014). Kaplan–Meier analysis demonstrated significantly lower event-free survival in patients with PT, with higher rates of all-cause mortality and post-discharge adverse events compared with patients with an emotional or no trigger (log-rank, *P* = 0.003; *Figure 2* and Supplementary data online, *Figure S1*), whereas no significant differences were observed according to the specific type of physical trigger (see Supplementary data online, *Figure S2*).

Univariable and multivariable predictors of the primary endpoints are reported in *Tables 2* and *3*.

**Table 2:** Predictors of all-cause mortality and post-discharge adverse events in univariable cox regression analysis

Variables	HR	95% CI	*P*-value
Age	1.005	[0.98, 1.02]	0.646
Sex	1.235	[0.59, 2.57]	0.574
Hypertension	1.605	[0.96, 2.66]	0.067
Dyslipidemia	0.895	[0.56, 1.41]	0.636
Obesity	1.066	[0.54, 2.08]	0.852
Smoke	0.871	[0.51, 1.47]	0.606
Diabetes	1.108	[0.58, 2.10]	0.755
CAD	1.673	[0.89, 3.11]	0.105
Typical chest pain	0.648	[0.40, 1.02]	0.066
Dyspnoea	1.794	[1.12, 2.85]	**0**.**014**
Emotional trigger	0.519	[0.31, 0.85]	**0**.**011**
No trigger	0.924	[0.56, 1.52]	0.755
Physical trigger	1.984	[1.25, 3.14]	**0**.**004**
Apical ballooning	1.257	[0.67, 2.34]	0.473
Mid-ventricular ballooning	1.126	[0.48, 2.61]	0.782
Basal ballooning	0.673	[0.16, 2.75]	0.582
Focal ballooning	1.187	[0.47, 2.95]	0.71
COPD	1.819	[0.90, 3.67]	0.095
Malignancies	1.273	[0.74, 2.16]	0.374
Neurological disease	2.182	[1.19, 3.97]	**0**.**011**
Psychiatric disesase	1.444	[0.73, 2.82]	0.285
Typical chest Pain	0.648	[0.40, 1.02]	0.066
Dyspnoea	1.794	[1.12, 2.85]	**0**.**014**
Duration of hospitalization	1.008	[0.98, 1.03]	0.526
T-wave inversion	0.986	[0.57, 1.69]	0.958
ST-segment elevation	0.863	[0.47, 1.56]	0.626
QT interval	0.997	[0.91, 1.03]	0.332
LVEF (per 1% increase)	0.980	[0.96, 0.99]	**0**.**035**
RVEF (per 1% increase)	0.965	[0.94, 0.98]	**0**.**002**
T2-STIR	0.944	[0.56, 1.58]	0.827
LGE	1.020	[0.54, 1.89]	0.94

Numbers in bold type indicate a significant difference.

CAD, coronary artery disease; COPD, Chronic Obstructive Pulmonary Disease; LGE, late gadolinium enhancement; LV, left ventricle; LVEF, left ventricular ejection fraction; RV, right ventricle; RVEF, right ventricular ejection fraction; STIR, short tau inversion recovery.

**Table 3:** Predictors of all-cause mortality and post-discharge adverse events in multivariable cox regression analysis

Variables	HR	95% CI	*P*-value
Dyspnoea	1.620	[0.99, 2.64]	0.054
Emotional trigger	0.871	[0.48, 1.57]	0.646
Neurological disease	2.125	[1.09, 4.14]	**0.027**
Physical trigger	1.758	[1.03, 2.98]	**0.037**
LVEF (per 1% increase)	0.990	[0.96, 1.01]	0.460
RVEF (per 1% increase)	0.982	[0.95, 1.01]	0.254

Numbers in bold type indicate a significant difference.

LVEF, left ventricular ejection fraction; RVEF, right ventricular ejection fraction.

In univariable Cox regression analysis, dyspnoea at presentation was associated with a higher risk of adverse outcomes (HR 1.794, 95% CI 1.12–2.85, *P* = 0.014), as were physical triggers (HR 1.984, 95% CI 1.25–3.14, *P* = 0.004) and neurological disease (HR 2.182, 95% CI 1.19–3.97, *P* = 0.011). Conversely, emotional triggers were protective (HR 0.519, 95% CI 0.31–0.85, *P* = 0.011). Impaired ventricular function also predicted worse outcomes, with each 1% increase in LVEF associated with lower risk (HR 0.980, 95% CI 0.96–0.99, *P* = 0.035) and each 1% increase in RVEF conferring an even lower risk (HR 0.965, 95% CI 0.94–0.98, *P* = 0.002).

In the Cox proportional hazards model including the three trigger categories and using emotional triggers as the reference, patients with PT showed a significantly higher risk (HR 2.31, 95% CI 1.32–4.05, *P* = 0.003), whereas patients with no triggers demonstrated a non-significant trend toward increased risk (HR 1.50, 95% CI 0.82–2.73, *P* = 0.188).

In multivariable Cox regression analysis adjusted for variables statistically significant in the univariable analysis, PT (HR 1.758, 95% CI 1.03–2.98, *P* = 0.037) and neurological disease (HR 2.125, 95% CI 1.09–4.14, *P* = 0.027) were independently associated with a higher risk of adverse outcomes. In the fully adjusted multivariable Cox regression model, which included variables statistically significant in the univariable analysis as well as key biological covariates, PT (HR 2.298, 95% CI 1.19–4.41, *P* = 0.012) remained independently associated with an increased risk of adverse outcome. *Supplementary data online, Table S1*.

## Discussion

The main results of the current multicenter study can be summarized as follows: (i) PT were identified in approximately one-third of TS patients; (ii) TS patients with PT exhibited lower troponin levels, higher inflammatory markers, and longer hospitalization compared with patients with emotional or no triggers; (iii) on CMR, PT was associated with less myocardial oedema and LGE, but higher rates of pericardial and pleural effusion; (iv) TS patients with PT experienced more frequent in-hospital complications and adverse long-term outcomes, including higher all-cause mortality and post-discharge adverse events; (v) in multivariable Cox regression analysis, PT was independent predictor of adverse outcomes.

In our cohort, PT were identified in approximately one-third of TS patients, a prevalence that closely mirrors findings from previous international registries. In the GEIST registry of 2,482 patients, 910 (36.7%) presented with an emotional trigger, 855 (34.4%) with a PT, and 717 (28.9%) with no identifiable trigger.^13^ Similarly, other multicenter studies have confirmed that PT represent a substantial proportion of TS presentations.^25,26^

TS patients with PT exhibited lower troponin levels but higher inflammatory markers and longer hospitalization compared with those with emotional or no triggers. The lower troponin release may suggest less direct myocardial injury, potentially related to sustained catecholamine elevation in PT, whereas emotional triggers typically induce a sudden catecholamine surge.^13,27^ Conversely, the significantly higher CRP values in PT patients likely reflects the systemic stress associated with acute illnesses or comorbid conditions acting as triggers.^28,29^ This systemic involvement may in turn contribute to the observed longer duration of hospitalization.^28^

CMR findings showed that PT patients had less frequent and less extensive myocardial oedema and LGE compared with those with emotional or no identifiable triggers. This supports the hypothesis that emotional stress induces a more abrupt catecholamine surge, leading to greater transient myocyte damage and interstitial oedema, while physical stressors are more often associated with systemic illness and haemodynamic compromise rather than direct cardiotoxic effects.^13,27^

Conversely, pericardial and pleural effusions were more common in patients with PT, likely reflecting the systemic inflammatory response and fluid overload.^30,31^

Additionally, our data demonstrated a higher rate of both in-hospital and post-discharge adverse events in patients with TS related to physical stress, consistent with previous studies.^3,13,26,27,32,33^

In the GEIST registry, patients with PT showed significantly higher rates of in-hospital complications (27.1% vs. 12.1% in emotional triggers) and higher long-term mortality (21.6% vs. 8.5%; both *P* < 0.001).^13^ Evidence from other large cohorts reinforces this high-risk profile: in the RETAKO registry, PT independently predicted 5-year mortality (HR 3.1; 95% CI 1.8–4.3; *P* < 0.001),^34^ while data from the Tokyo CCU Network demonstrated that medical illnesses acting as PT independently predicted all-cause death (OR 4.73; 95% CI 1.33–16.87).^33^ Similarly, the InterTAK Registry identified PT, along with acute neurological or psychiatric disorders, elevated troponin levels, and reduced left ventricular ejection fraction on admission, as independent predictors of in-hospital complications.^3^

Nevertheless, the mechanisms underlying these differences remain incompletely understood. The increased complication rate in patients with PT likely reflects, at least in part, the severity of the underlying critical illness, which not only precipitates TS but also contributes to adverse outcomes and mortality. Importantly, significant differences in cardiac biomarkers and CMR characteristics were still observed between trigger groups. These findings support a theoretical framework in which the type of trigger shapes both the acute myocardial response and the subsequent clinical trajectory. Emotional stress tipically induces a sudden catecholamine surge that predominantly affects the myocardium, leading to transient but pronounced oedema, whereas physical stressors are associated with sustained adrenergic activation, systemic inflammation, and metabolic stress. This persistent catecholamine drive may amplify vulnerability to complications and worsen long-term prognosis.^27,34^ Recognizing this heterogeneity has important clinical implications for risk stratification and management, underscoring that patients with physically triggered TS may benefit from more intensive monitoring and proactive management strategies both during hospitalization and in the outpatient setting.

### Limitations

This study has several limitations. First, its retrospective design restricted the availability and completeness of certain clinically relevant data. Second, although the registry was multicenter, the relatively small number of TS patients with documented PT may limit the generalizability of the findings. Third, CMR examinations were not consistently performed at the time of symptom onset, which may have reduced the sensitivity for detecting transient features of TS. Importantly, patients with PT may represent a population with more severe systemic illness, such as acute infections, major surgery, or neurological events. Consequently, the observed worse outcomes in this subgroup could reflect the underlying severity of the precipitating condition rather than the TS phenotype itself, introducing potential reverse causality. While our study was not powered to perform a formal sensitivity analysis excluding these cases, this limitation should be considered when interpreting the results.

Nevertheless, these limitations notwithstanding, the study provides valuable preliminary insights into the potential significance of PT in TS and underscores the need for larger, prospective, multicenter studies with standardized imaging protocols to validate and expand these observations

## Conclusion

In this multicenter, international study, patients with TS triggered by PT exhibited a distinct clinical and imaging phenotype, characterized by higher in-hospital complication rates and worse long-term outcomes compared with those with emotional or no triggers. Consequently, these patients warrant closer surveillance and more aggressive management strategies.

## Supplementary Material

jeag017_Supplementary_Data

## Data Availability

The data will be shared on reasonable request to the corresponding author, who will submit the request to the EVOLUTION group.

## Appendix

The 5VOLUTION group: Cosimo Agrimi, Luca Arcari, Francesco Balata, Leon Bischoff, Alfredo Blandino, Federica Catapano, Riccardo Cau, Federica Ciolina, Alberto Clemente, Jean Nicolas Dacher, Tommaso D’Angelo, Fabrizio D’Ascenzo, Antonio Esposito, Riccardo Faletti, Nicola Galea, Marco Gatti, Massimo Imazio, Costanza Lisi, Julian Luetsken, Maria Francesca Marchetti, Gloria Marras, Antonella Meloni, Roberta Montisci, Giuseppe Muscogiuri, Francesco Negri, Anna Palmisano, Giacomo Pambianchi, Alessandro Pinna, Laura Pistoia, Francesco Pisu, Gianluca Pontone, Luca Saba, Normant Sebastien, Giulio Antonino Strazzarino, Alessandra Volpe, Benedetta Volpi.
