# Supplementary material for: Physical triggers in takotsubo syndrome: a high-risk phenotype? insights from the eVOLUTION registry
Source: Eur Heart J Cardiovasc Imaging. 2026 Feb 5;27(4):757–65. doi: 10.1093/ehjci/jeag017 (PMC13021275; doi:10.1093/ehjci/jeag017)
Supplement: jeag017_Supplementary_Data [file jeag017_supplementary_data.docx]

**Supplemental material**

**Supplemental table 1**

| **Variables** | **HR** | **95% CI** | **p-value** |
| --- | --- | --- | --- |
| **Dyspnea** | 1.838 | [1.05, 3.36] | **0.048** |
| **Emotional Trigger** | 0.894 | [0.43, 1.81] | 0.757 |
| **Neurological disease** | 1.659 | [0.78, 3.50] | 0.184 |
| **Physical Trigger** | 2.298 | [1.19, 4.41] | **0.012** |
| **LVEF (per 1% increase)** | 0.996 | [0.97, 1.04] | 0.734 |
| **RVEF (per 1% increase)** | 0.963 | [0.92, 1.05] | 0.082 |
| **Age** | 1.002 | [0.97, 1.03] | 0.901 |
| **Sex** | 1.057 | [0.43, 2.58] | 0.904 |
| **Troponin T** | 1.000 | [0.99, 1.01] | 0.140 |
| **C-reactive Protein** | 1.002 | [0.95, 1.05] | 0.638 |
| **Duration of hospitalization** | 1.010 | [0.98, 1.04] | 0.482 |

**Supplemental Table 1**: Predictors of all-cause mortality and post-discharge adverse events in the full multivariable Cox regression model, which included all variables with p < 0.05 in univariable analysis and additional variables of biological significance.

Numbers in bold type indicate a signicant difference.
Abbreviations: LVEF, left ventricular ejection fraction; RVEF, right ventricular ejection fraction.

**Supplemental figure 1**

**

**

**Supplemental figure 1:** Kaplan–Meier curves comparing patients with Takotsubo syndrome with and without physical triggers. Panels show (A) all-cause mortality and (B) post-discharge adverse events, illustrating the higher event rates in patients with physical triggers.

**Supplemental Figure 2**


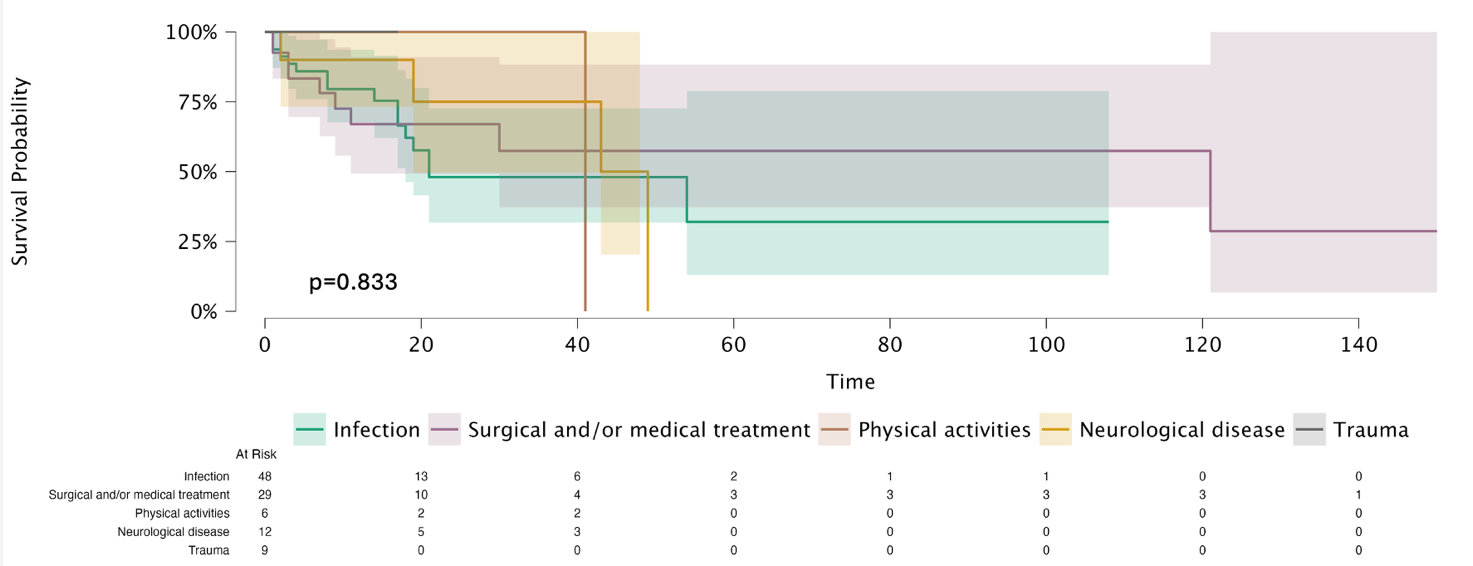


**Supplemental figure 2:** Kaplan–Meier curves illustrating long-term outcomes in patients with Takotsubo syndrome according to the specific type of physical trigger. No significant differences in all-cause mortality or post-discharge adverse events were observed among the different types of physical triggers.
